# Identifying Priority Health Conditions, Environmental Data, and Infrastructure Needs: A Synopsis of the Pew Environmental Health Tracking Project

**DOI:** 10.1289/ehp.7147

**Published:** 2004-08-03

**Authors:** Jill Litt, Nga Tran, Kristen Chossek Malecki, Roni Neff, Beth Resnick, Thomas Burke

**Affiliations:** ^1^University of Colorado Health Sciences Center, Department of Preventive Medicine and Biometrics, Denver, Colorado, USA; ^2^Exponent Consulting, Washington, DC, USA; ^3^The Johns Hopkins Bloomberg School of Public Health, Department of Health Policy and Management, Baltimore, Maryland, USA

**Keywords:** biomonitoring, disease surveillance, environmental health indicators, environmental public health tracking, exposure, health policy, information systems

## Abstract

In this article we describe the methodologic approaches of the Pew Environmental Health Commission at the Johns Hopkins Bloomberg School of Public Health used to identify priority environmental health conditions and develop recommendations to establish a national environmental public health tracking network. We present the results of a survey of public health and environmental practitioners to uncover state and local health tracking needs and priorities. We describe the steps that combined the findings from the state and local health tracking survey and a review of the state of the science on environmental impacts on health to identify priority health end points. Through an examination of national health and health care databases, we then describe trends and public health effects of those diseases that may be linked to the environment. Based on this analysis, respiratory diseases and neurologic diseases are recommended as priorities for tracking. Specific end points recommended for tracking include asthma and chronic respiratory diseases, and chronic neurodegenerative diseases such as multiple sclerosis. Based on trends in reported prevalence, consideration should also be given to developmental disabilities, reproductive disorders, and endocrine/metabolic disorders. Strengthening of current efforts to track cancer and birth defects should also be included as components of a nationwide health tracking network. Finally, we present the recommendations for environmental public health tracking. These recommendations provided the groundwork for the development of the Centers for Disease Control and Prevention’s National Environmental Public Health Tracking Progam that now includes 21 states, three cities, and three academic centers throughout the nation.

The new millennium has brought unprecedented challenges and opportunities for the advancement of public health. Scientific breakthroughs provide new insights into the genetic basis of health and disease, while analytical advances allow the identification and measurement of previously unrecognized threats. The translation of this new knowledge to effective prevention will require new approaches to evaluating the interaction of health and environment. Despite the many advances in identification, measurement, and control of contaminants in the environment, fundamental questions about the role of environmental exposures in human disease remain unanswered. This information gap includes a number of chronic diseases and conditions that may be increasing in prevalence, including asthma, neurologic disorders, developmental disabilities, and even diabetes.

The Pew Environmental Health Commission at the Johns Hopkins Bloomberg School of Public Health was established in 1998 to develop a blueprint to rebuild the nation’s public health defenses against environmental threats, including the ability to track hazards, environmental exposures, and health outcomes. Greater capacity in tracking can help investigators *a*) identify populations at risk and respond to outbreaks, clusters and emerging threats; *b*) establish the relationship between environmental hazards and disease; *c*) guide intervention and prevention strategies, including lifestyle improvements; *d*) identify, reduce, and prevent harmful environmental risks; *e*) improve the public health basis for policymaking; *f* ) enable the public’s right to know about health and the environment; and *g*) track progress toward achieving a healthier nation and environment.

In 1999–2000 the Pew Environmental Health Commission’s Tracking Project (Pew Environmental Health Commission 2000) conducted an extensive evaluation of the national environmental public health infrastructure and found a fundamental information gap in our understanding of the relationship between environmental exposures and the health of the public. In this article, we present a series of methodologic approaches used to identify priority health conditions for environmental health tracking and develop the recommendations of the Pew Commission.

## Methods

We describe a multistep approach to address the goals of the Pew Commission’s tracking project. First, we describe the development and implementation of a tracking survey administered to public health and environmental practitioners. Second, we describe steps taken to inform the selection of priority health end points for environmental health tracking, which included a synthesis of review articles on environmental health indicators and an examination of existing national environmental emissions, health, and health care databases to understand trends in environmentally related health end points. Finally, we discuss the process for translating the findings from our research to Pew Commission recommendations.

### State and local survey.

State and local public health practitioners were surveyed by telephone to obtain information about their environmental public health tracking programs. Questions focused on organization, data collection and use, financial and technical resources, barriers, and priorities. State respondents (*n =* 49) were primarily environmental epidemiologists. Local respondents (*n =* 21 counties) were mostly health department directors who had expressed interest in environmental public health, suggesting a best-case scenario. Representatives included all 12 local representatives on the National Association of City and County Health Officials (NACCHO) Environmental Health and Prevention Advisory Committee in addition to 10 local health departments randomly selected from a NACCHO list of 133 local health departments that identified environmental health as an interest area. Surveys were conducted from March to September 2000. A follow-up survey is under way in 2004.

### Identification of priority health conditions.

Tracking the health of a population is an essential component of effective public health practice. However, given the many gaps in our current understanding of the role of the environment in disease, the Pew Commission was faced with a fundamental question: “What health end points should be tracked?” To address this question, we developed an approach to evaluate available national data to identify health end points that may be appropriate for inclusion in a national environmental health tracking network.

#### Step 1—Literature review of environmental health indicators.

We evaluated the scientific literature to identify specific health effects that have been related to environmental exposures and that may serve as environmental health indicators. These indicators can be useful in providing measures of population health that can be related to environmental exposures, thereby providing a public health yardstick for measuring environmental progress. This review included published work by health and environmental agencies identifying diseases or health end points that have a possible link to environmental exposures.

#### Step 2—Connecting environmental releases and health conditions.

We used the Environmental Defense Scorecard analysis that provides listings of chemicals that impact human health (Environmental Defense 2000; http://www.scorecard.org). These listings were derived from toxicologic and epidemiologic studies and information from regulatory agencies. The U.S. Environmental Protection Agency (EPA) Toxics Release Inventory (TRI; http://www.epa.gov/tri/) served as the starting point for the chemical listings. The Scorecard ranking exercises combined the TRI data with information on potential health hazards of these substances. These reports account only for pollution from industrial facilities that reported to TRI in 1997 (the most recent year available for our analysis) and include only the 644 chemicals covered by TRI at that time.

#### Step 3—Examination of national health databases.

On the basis of Scorecard’s list of substances, related broad health categories, and the literature review, we analyzed available health outcome data at the national level for selected end points to identify those with high or increasing prevalence or those responsible for heavy utilization of health care. There is virtually no comprehensive national tracking of noninfectious diseases (except cancer). However, four National Center for Health Statistics (NCHS) national survey data sets including the National Health Interview Survey (NHIS), the National Ambulatory Medical Care Survey (NAMCS), the National Hospital Ambulatory Medical Care Survey (NHAMCS), and the National Hospital Discharge Survey (NHDS) were examined for this analysis and provided useful insights into some of the end points identified in steps 1 and 2 (NCHS 2000; 2004a; 2004c; 2004d).

Initially the analysis was intended to be limited to environmental health outcomes that are clinically observable and classifiable by the *International Classification of Diseases,* 9^th^ Revision (ICD-9) codes and that have been linked to identifiable exposures to environmental agents (Health Care Financing Agency 1998). Because of the limitations of the available survey and emerging interests in a broader range of health end points, the analysis included a number of end points without known environmental causes. Inclusion of a disease in this analysis is not meant to imply environmental etiology. Given the present limitations of knowledge, even in most cases where an environmental exposure has been shown to contribute to the development of adverse effects, it is not possible to quantify the proportion of risk attributed to the environment.

The NHIS provides valuable national-level information on the prevalence of and trends for some key health outcomes. Published NHIS estimates for both chronic and acute conditions are available as far back as 1984. Categories of end points are grouped by ICD-9 codes in a process known as the NHIS recode (NCHS 2000). The 1997 survey was used to estimate rates of childhood disorders previously not covered by NHIS and adult conditions for comparison to earlier years. These data were obtained from the NCHS web site (NCHS 2004c), imported into SAS (version 8.0; SAS Institute, Inc., Cary, NC), and were used to develop estimates separately for children (< 18 years of age) and adults (18 years +) using the appropriate weights.

Depending on the disease category, these groupings may or may not be specific to environmental health end points. In addition, these categories may include a limited number of end points and may provide a misleading estimate of the prevalence of disease in the population. For example, the NHIS grouping for neurologic diseases includes migraine headaches but excludes diseases of growing interest such as Alzheimer and Parkinson diseases, thus resulting in an underestimation of prevalence of neurologic diseases in the population. This is a major limitation of the NHIS data set when evaluating disease trends that may be influenced by environmental exposures. Conversely, respiratory diseases are captured more accurately by the NHIS recode. The disease prevalence and incidence rates give a better assessment of respiratory conditions with environmental etiology such as asthma and chronic obstructive pulmonary disease.

In addition to disease trends, we reviewed health care information from the 1996 NHDS, NAMCS, and NHAMCS. Estimates from the NHDS were obtained for 1996 (NCHS 2004d). This source provided estimates of the number of discharges for individual ICD-9 codes, already weighted to the U.S. population. To estimate rates, U.S. census data were used to obtain the total U.S. population numbers for 1996, as well as population estimates by age, region, and sex (Population Estimates Program 1999a, 1999b).

Estimates from the 1996 NHAMCS and the 1996 NAMCS were obtained from public data sets available from the NCHS web site (NCHS 2004d). For analysis, the data sets were input into SAS, version 8.0. To estimate the number of visits for a particular ICD-9 code, that code was selected as the principal diagnosis and a weighted PROC FREQ command was used. To estimate the proportion of office visits because of a particular condition, the total number of office visits was used as the denominator. To estimate visit rates, the denominator was the 1996 U.S. population, obtained from U.S. census publications (Population Estimates Program 1999a, 1999b). Once weighted, these estimates represented U.S. population visits to physicians’ offices, hospital emergency departments, and hospital outpatient clinics for medical care.

### Environmental summit.

As a follow-up to the previous research steps, the Pew Commission, in partnership with the Association of State and Territorial Health Officials, NACCHO, and the Public Health Foundation, hosted over 50 federal, state, and local environmental health leaders for 2 days to develop recommendations to establish and implement national environmental health tracking.

## Results

### State and local survey.

#### Data collection and use: hazards, exposures, and health outcomes.

Environmental public health tracking programs were diverse. In general, much of the hazard tracking was handled by environmental agencies, while public health agencies, especially at the state level, most frequently handled health outcome tracking. Exposure tracking was rare with the exception of lead (81% of state public health agencies) and personal air monitoring data (one-fourth of state health agencies.) States reported the following for health outcome tracking activities. Most states tracked cancer (94%), infectious outbreaks (80%), and birth defects (69%), but many did not track asthma (55%) and few tracked emerging environmental health concerns such as developmental disabilities (16%), learning disorders (12%), or autoimmune diseases (8%). Some of the larger local public health agencies collected significant quantities of tracking data, but many locals relied on other agencies to collect data, often seeing their role more in dissemination and follow-up investigations.

#### Coordination.

To improve their effectiveness and leverage limited capacities, state and local officials worked with other federal, state, and local agencies on environmental public health issues. Often, state public health departments facilitated relationships between federal and local agencies. Given the coordination challenges, 53% of states and 81% of local departments designated lead individuals for overseeing tracking activities.

#### Financial and technical resources.

Among states, major sources of funding for environmental public health tracking were state general funds (81% of states), federal funds from grants and cooperative agreements with the Centers for Disease Control and Prevention (CDC), the Agency for Toxic Substances and Disease Registry (ATSDR), the U.S. EPA and the National Institute for Occupational Safety and Health (55, 45, 34 and 23%, respectively), prevention block grants (32%), and fee-based regulatory programs (21%). At the local level, 90% of departments received local general funds, and 86% received funds from fees and permits to support their tracking activities. Forty-eight and 38% of departments, respectively, received state department of health and environment funding, and only 33% received federal funding. (Some federal funds may have been channeled through state programs.)

From a technologic perspective, 81% of the states indicated that health and environmental data were electronically available for internal use, and 69% made health data available to the public in limited electronic formats and geographic scales. Local public health departments varied widely in their access to current computer software and/or hardware, and in their ability to use technology to its full extent. The lack of standardization in state, county, and other data systems hindered the ability to access and interpret data.

#### Environmental public health tracking priorities*.*

States revealed a diverse set of priorities for environmental public health tracking. Drinking water, metals, food protection, asthma, cancer, and for some states, the need to establish basic tracking capacity and assure room for flexible approaches to tracking were top priorities. At the local level, top priorities, as reflected by spending, were food protection, waste management, and water and air quality. Further priority needs included indoor air, bioterrorism and emergency response, and land use issues.

#### Barriers.

At both the state and local levels, the greatest identified barrier to effective environmental public health tracking was scarce financial resources, followed by the related need for staff. Respondents wanted flexible funding strategies and increases in staff and expertise. Local agencies were particularly concerned about unfunded tracking recommendations or requirements. Other important barriers included a lack of political will, limited reporting requirements, lack of established databases, and their own organizational structure. Further, some wanted guidance on tracking system design, implementation, and priorities.

### Identification of priority health conditions results.

#### Step 1—Literature review.

Through the literature review, we identified key health outcomes partially determined by environmental exposures. In 1993 the ATSDR authored a report (Lybarger and Spengler 1993) that identified seven broad groupings of health conditions where research is needed to elucidate the exposure–disease relationship. These conditions included respiratory diseases, neurologic disorders, congenital anomalies, reproductive disorders, kidney diseases, immune disorders, and cancer.

Rios et al. (1993) presented a review of research on biologic susceptibility of minority populations to environmental pollutants that may result from genetic makeup, occupation, pre-existing health conditions, exposure to mixtures of pollutants, substance abuse, unemployment, and other social inequalities in health care, education, and political power. The outcomes of concern included respiratory diseases (e.g., asthma and chronic obstructive pulmonary disease); chronic liver disease and cirrhosis; heart disease; sickle cell anemia; kidney disease; and endocrine disorders including diabetes mellitus.

In 1994 Silbergeld (1994) identified three health conditions whose etiologies remain largely unexplained but where environmental exposures are implicated. She suggested that epidemiologic research on asthma, low birth weight, and neurodegenerative diseases was central to improving our environmental health policies and their subsequent benefits.

Turning to the international literature, Kjellstrom and Corvalan (1995), as part of the World Health Organization project HEADLAMP (Health and Environment Analysis and Indicators for Decision-Making) identified seven indicators that would serve as monitoring tools for the United Nations initiative on sustainable development. These indicators included asthma; skin disorders; aplastic anemia; birth defects, including congenital anomalies and low birth weight; spontaneous abortions and cancer.

Regarding the connections between air pollution and mortality, particularly respiratory and cardiovascular mortality, Kelsall et al. (1997) provided one example of the growing body of epidemiologic work establishing this association. They presented scientific evidence that particular health conditions increase human susceptibility to environmental pollutants.

The U.S. Department of Health and Human Services (U.S. DHHS) Healthy People initiative regularly tracks the public’s health and has set objectives for quantifiable reductions in disease and disability over the past 20 years. Community health and environmental health indicators drawn from Healthy People objectives included asthma and chronic obstructive pulmonary disease, chronic liver disease and cirrhosis, heart disease, methemoglobinemia, congenital anomalies, low birth weight, developmental disabilities, kidney diseases, cancer and endocrine disorders including diabetes mellitus (U.S. DHHS 1998).

#### Step 2—Connecting environmental releases and health conditions.

Based on the reviews of the Scorecard data and related health categories, we found that substances with potential respiratory effects were the highest category of releases in 1997 and this continues to be the highest ranking category based on 2001 TRI data. Neurologic, skin, gastrointestinal, and liver toxicants were next highest categories in total pounds released. Based on 2001 data, these categories have shifted slightly, with skin disorders replacing neurologic conditions in the second highest category and gastrointestinal diseases moving up to the third category. Other categories included cardiovascular, developmental, and reproductive effects.

#### Step 3—National health outcome and health care databases.

**Figure 1:**
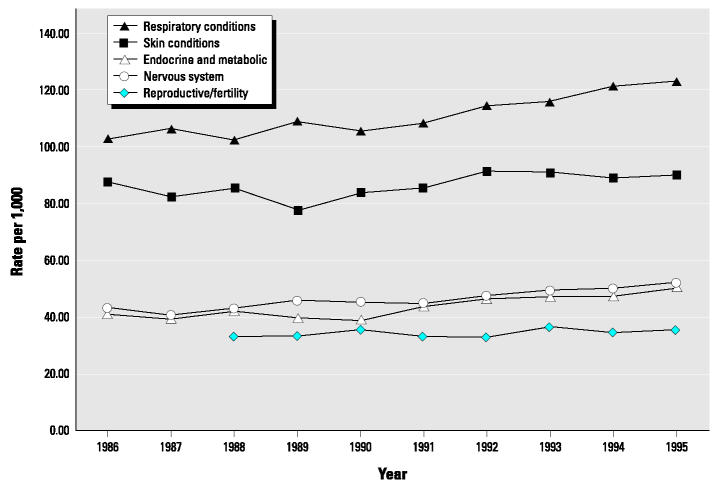
Self-reported prevalence for selected categories of disease. Data from National Health Interview Survey, 1986–1995 (NCHS 2004c). Changes in reproductive and fertility outcomes reflect years 1988–1995.

Figure 1 shows 10-year trend data for the self-reported prevalence of a number of broad categories of health conditions, including respiratory conditions, skin diseases, neurologic disorders, reproductive and fertility conditions, endocrine and metabolic conditions. Specifically, endocrine and metabolic disorders show the greatest increase (21.7%), followed by neurologic (20%) and respiratory diseases (20%). As previously noted, because of the NHIS recodes, these broad categories of health conditions are combinations of conditions, reflecting end points with and without known environmental etiologies.

We also evaluated trend data over the same time period within specific health categories. For example, within respiratory conditions, asthma rates increased by 38.6% and chronic bronchitis increased by 15.3%. For endocrine and metabolic diseases, thyroid disorders increased by 36.3% and diabetes mellitus increased by 19.1% over this time period. For neurologic diseases, multiple sclerosis increased by 21.2% and migraine headaches increased by 26%. Within the reproductive health category, prostate diseases (noncancer including hyperplasia, inflammation) increased by 48% and disorders of female reproductive organs (e.g., ovarian cysts, disorders of the uterus and cervix) increased by 28.6%.

We also summarized the number of hospital discharges, emergency department visits, hospital outpatient care visits, and doctor office visits for six broad groupings of health outcomes, including lung and respiratory conditions, neurologic conditions, reproductive and fertility conditions, blood disorders, liver disease, and cardiovascular disorders. Specific disease end points within these broad classifications were included where information was available.

Cardiovascular diseases required the most health care resources, including over 48 million doctor visits and 4.6 million hospitalizations. Although pollution exposures have been indicated for some types of cardiovascular diseases, many other environmental risk factors, including lifestyle and obesity, have been implicated as contributing causes. Lung and respiratory diseases (33.6 million doctor visits and over 3 million emergency department visits) and neurologic conditions (8.7 million doctor visits) also required large amounts of health care services. Of all the lung and respiratory health conditions resulting in utilization of the health care system, asthma and chronic bronchitis accounted for the largest proportion of hospital, emergency department, outpatient and doctor visits in 1996. Among the endocrine conditions, diabetes resulted in the most health care use (over 15 million physician visits). Of the neurologic conditions requiring health care, a relatively small fraction was due to neurodegenerative diseases such as senility, cerebral degeneration, and Alzheimer and Parkinson diseases. However, these diseases have a devastating impact on the quality of life and require care that may not be measured by these surveys.

## Environmental Summit—Stakeholder Priorities

Summit participants developed specific recommendations for improving the national environmental health infrastructure and capacity for tracking. They did not identify specific exposures or health end points for tracking. Recognizing current scientific limitations concerning the role of environment in disease and state and regional differences in environmental health priorities, they recommended a flexible tiered approach. Recommendations included

- National tracking of high priority exposures and health outcomes
- A sentinel network to identify emerging hazards
- A coordinated network of pilot regional, state, and local tracking programs
- A supportive research program to guide and evaluate tracking progress

These recommendations provided the basis for the Pew Commission recommendations that have shaped the development of the CDC Environmental Public Health Tracking Program.

## Conclusions

The surveys of state and local public health department officials provided baseline data on the state of environmental public health tracking in 2000. The surveys found great variety in tracking organization, functions, and resources among state and local health departments. The overall infrastructure for environmental public health tracking lacked adequate support, personnel, coordination, and data resources. Collection of these baseline data will provide an opportunity to evaluate the impact of the new tracking initiatives and resources as well as increased focus on preparedness since 2000.

The priority health condition analysis identified a number of limitations that must be addressed. These findings are constrained by available epidemiologic and toxicologic data and are driven by high-volume chemical release reporting under the TRI. Multiple health effects can be associated with an individual toxicant, and complex interactions between toxicants can further affect human health. Nevertheless, this approach provided the Pew Commission with a starting point for identifying the categories of health end points to be considered for tracking. Given the large amount of toxic pollutants released, there is a need to improve the tracking of population exposures and look for any evidence of adverse health impacts.

The end points identified through the literature represent conditions for which environmental exposures have been implicated or are pre-existing health conditions that may be exacerbated by exposure to environmental pollutants. The end points also reflect agency priorities, including ATSDR’s seven broad categories of priority health conditions and the U.S. DHHS Healthy People objectives for community and environmental health indicators (U.S. DHHS 1998 U.S. DHHS 2000). Although this list of health end points is culled from numerous sources with diverse criteria, the categories and end points show a general convergence that can shape priorities for the developing tracking network.

An examination of available national survey data indicated that the reported prevalence of several categories of disease potentially related to the environment has been increasing. Between 1986 and 1995 the largest increases were reported in endocrine and metabolic disorders (21.7%), followed by neurologic (20%) and respiratory diseases (20%). Reproductive disorders also increased during this time (7.3%).

Available data on health care utilization for these outcomes indicate that cardiovascular disease requires the greatest use of health care, with respiratory (over 33 million doctor visits and 3 million emergency department visits) and neurologic diseases (over 8 million doctor visits) also requiring large amounts of health care services.

Based on this analysis of the weight of evidence and trends in health outcomes and impacts, respiratory diseases and neurologic diseases are recommended as priorities for tracking. Specific end points recommended for tracking include asthma and chronic respiratory diseases, and chronic neurodegenerative diseases such as multiple sclerosis. Based on increasing trends in reported prevalence and the potential for environmental exposures to increase population risks, consideration should also be given to developmental disabilities, reproductive disorders, and endocrine and metabolic disorders. Strengthening of current efforts to track cancer and birth defects should also be included as components of a nationwide environmental health tracking network.

The role of the environment in the etiology of these health outcomes remains unknown. Identification of specific outcomes as tracking priorities should not be interpreted as an implication of environmental causality. However, the increasing incidence and prevalence of a number of diseases with potential links to environmental exposures underscores the need for improved tracking to increase our understanding of risk factors, identify populations at high risk, inform priorities for research, and develop coordinated prevention efforts.

## Discussion

Advances in hazard identification, exposure assessment, health outcome data collection, and information technology provide mechanisms for advancing tracking and improving our understanding of the environment and health. These advances, coupled with deep public concern, provide a window of opportunity to strengthen the national infrastructure for environmental health information, expand public access to this important information, and protect the privacy of individuals. New technologies in biomonitoring have the potential to transform the nation's capacity to track exposures to pollutants and understand their impacts on health. Advances in communication and information technology have expanded opportunities for public access and given us new tools to analyze, map, and disseminate health data. New technology also can improve safeguards to protect the confidentiality of identifiable personal health information.

Building on the findings and recommendations of the Pew Commission, CDC developed the Environmental Public Health Tracking Program (NCEH 2004). CDC was allocated funds in fiscal years 2002 and 2003 for $14.2 and $14.6 million, respectively, to fund 24 state and local health departments and three public health schools to *a*) build environmental public health capacity, *b*) increase collaboration between environment and health agencies, *c*) identify and evaluate environmental and health data systems, *d*) build partnerships with nongovernmental organizations and communities, and *e*) develop model systems that link environmental and health data and that other states or localities can use. Three public health schools are funded to support state and local health departments and investigate possible links between health effects and the environment (NCEH 2004).

In addition, CDC together with the Center for State and Territorial Epidemiologists (CSTE) have developed a set of environmental public health indicators that can be used to assess baseline status and trends and build core surveillance capacity in state and local agencies (CSTE 2004). The U.S. EPA has also contributed to the national capacity for tracking by producing the Draft EPA Report on the Environment (U.S. EPA 2003), which provides information on the status of and trends in environmental conditions and their effects on human health and the nation's natural resources. These indicator initiatives provide summary measures of environmental health relationships that are fundamental to future environmental tracking efforts (U.S. EPA 2003).

Beyond these indicator programs, the CDC’s biomonitoring program and “National Report on Human Exposure to Environmental Chemicals” are enhancing our abilities to measure environmental chemicals in the human body (NCEH 2003). The data are collected as part of the National Health and Nutrition Examination Survey (NCHS 2004b), and specimens are analyzed as part of the CDC biomonitoring program. Between 2001–2003 the number of chemicals being measured and reported expanded from 27 to 116. The samples provide estimates of population exposures to key contaminants of concern and begin to fill a critical gap in our ability to link exposure and health outcome data (NCEH 2003).

Beyond strengthening the science and supporting data, efforts are under way to better link environment and health, including new relationships and collaboration between environment and health agencies. This integrated thinking brings new understandings and approaches to improve environmental protection efforts, to better characterize and control sources through public health surveillance, and to understand the links between adverse exposures and health effects.

The “building blocks” of knowledge provided by a nationwide environmental public health tracking network will enable scientists to answer many of the troubling questions we are asking today about what is making us sick. The result will be new prevention strategies aimed at reducing and ultimately preventing many of the chronic diseases and disabling conditions that afflict millions of Americans.
